# Poster 2011: A DNA vaccine immunotherapy for japanese red cedar allergy

**DOI:** 10.1186/1939-4551-7-S1-P23

**Published:** 2014-02-03

**Authors:** Lawrence Weiner, Bruce Mackler, Bill Hearl, David Fitz-Patrick

**Affiliations:** 1 Immunomic, Rockville, MD, USA; 2 East west research, Honolulu, HI, USA

## Methods

A Phase I study was conducted with *i* plasmid DNA vaccine, containing the CryJ2 allergen from Japanese Red Cedar inserted into the Lysosomal Associated Membrane nucleic acid sequence. Previouslyy sensitized Japanesei, were consented in English/Japanese, received four doses and assessed over 132 days for the safety of this vaccine. Group 1 contained non-sensitive and Groups 2 & 3 sensitive subjects as defined by +/- skin test to JRC, Mountain cedar and CryJ2 allergens. All subjects received 4 doses at 14 day intervals.

Safety data through 132 days after the 1st vaccination showed 85% of the adverse events (AE’s) were mild, mainly injection site erythema, swelling and pain, the majority occurring in Group 3.t All AEs were of a transient nature, requiring no medical attention. CryJ2-LAMP-Vax did not induce IgE CryJ2 specific titer changes, nor CryJ2 specific IgG titers. Prior to each vaccination and at 72 and 132 days, subjects tested negative for anti-LAMP antibodies. These clinical and biomarker data suggested that the plasmid vaccine was safe.

## Results

At 132 days, the skin prick tests indicated that the CryJ2-LAMP-Vax converted 10 of 12 subjects’ JRC positive skin test reactions to negative, with a similar conversion pattern in 6/11 Mountain cedar skin test positive; Mountain cedar Jun a 2 allergen shares a 91% homology with CryJ2. These subjects presumptively were sensitive to the Jun a2 allergen, 91% homologous to CryJ2.

## Conclusions

The most striking observation at Day 132 was the conversion of positive skin test reactions to unrelated allergens-Southern Grass mix, Western Ragweed mix, Southern California Tree Mix and Dust Mite Mix, to negative at day 132. In Groups 2 & 3, the conversion was 10/17 subjects. The vaccinated non-CryJ2 sensitive subjects exhibited no skin test conversions. These skin test conversions from positive to negative at day 132 for allergens unrelated to CryJ2, possibly represents a general T cell immune response due to the DNA vaccine.

The Phase I clinical data and laboratory results support the conclusion that vaccination with CryJ2-LAMP-Vax is safe and changes the immune status. The majority of AE’s were mild skin injection reactions, primarily in the high dose /Group 3. There were no anaphylactic reactions from the vaccination. The lack of any significant IgE anti-CryJ2 responses in both vaccinated non-atopic sensitive and atopic sensitive subjects indicated a conversion of the immune status to CryJ2 from a Th2 into a Th1 response. The skin test conversions of 21 subjects JRC/MC/CryJ2 skin tests to negative at day 132, suggests that the DNA-LAMP vaccine modulated the immune system. Further, it is suggested that the positive to negative skin test conversions for allergens unrelated to CryJ2 specificity, possibly represents a general bystander T cell regulatory response.

